# Far-Contralateral Oblique (FCO) Sacroiliac Joint Injection: Description of a Novel Technique

**DOI:** 10.1155/2022/3312589

**Published:** 2022-08-22

**Authors:** David W. Lee, Patrick Buchanan, Shashank Vodapally, Christopher James, Jack Diep

**Affiliations:** ^1^Fullerton Orthopedic Surgery Medical Group, Fullerton, CA, USA; ^2^Spanish Hills Interventional Pain Specialists, Camarillo, CA, USA; ^3^Michigan State University, Department of Physical Medicine & Rehabilitation, Lansing, MI, USA; ^4^University of KY, Department of Physical Medicine & Rehabilitation, Lexington, KY, USA; ^5^Lakeside Spine & Pain, Lake Havasu, AZ, USA

## Abstract

Sacroiliac (SI) joint arthropathy is the primary pain generator in approximately 15–25% of patients with axial low back pain and traditionally diagnosed with >50% pain reduction following an intra-articular injection localized to the inferior 1/3 of the SI joint. The conventional technique for accessing the SI joint encompasses a posterior approach with fluoroscopic guidance at 10–20⁰ contralateral oblique angulation, and minor adjustments to this approach have been implemented with varying degrees of success. The authors present a novel technique for SI joint injection, infiltrating the middle third of the joint through an alternative far-contralateral oblique (FCO) approach, angulation between 20–40⁰. This approach theoretically endows easier access to the SI joint and at the very least provides another option for interventionalists in the diagnosis and treatment of sacroiliac joint pain. It can also be utilized to determine if a patient is a candidate for posterior percutaneous SI joint fusion. The authors sought to document this approach to ensure that it was both reproducible and safe, while recognizing the need for future studies.

## 1. Introduction

Axial low back pain (LBP) has been one of the primary causes of years lived with disability, and it continues to be an expanding burden on health systems as the global population continues to grow [1, 2]. Though some patients experience LBP originating from facet mediated and vertebrogenic pain, about 15–25% of patients with LBP have pain originating primarily from the sacroiliac joint (SIJ) [3]. As the treatments offered for SIJ mediated pain continue to evolve, appropriately diagnosing this diarthrodial joint as the primary pain mediator becomes even more critical. Obtaining a complete history, evaluating prior images, and performing a thorough physical examination evaluating the SIJ and surrounding structures are the initial steps to this process. If the SIJ is the presumed pathology, a diagnostic intra-articular SIJ injection with local anesthetic is performed and the diagnosis is further elucidated with greater than 50% pain relief, pinpointing the SIJ as the etiology for the LBP [4]. Traditionally, the intra-articular SIJ injections are targeted towards the inferior 1/3 of the SIJ. Due to patients' variable anatomy and pathological changes that occur with time, accessing the joint from this area may be challenging.

The use of a far-contralateral oblique approach has been recently adopted in use of posterior percutaneous allograft placement [5]. The use of sacroiliac joint intra-articular joint injections with the same approach requires further exploration and description. The procedure is detailed with the purpose of illustrating a novel but practical alternative approach for sacroiliac joint injections and to describe a reproducible method of performing the procedure.

### 1.1. Anatomy

The sacroiliac joint is a diarthrodial joint that plays a critical role in transmitting vertical forces between the lower extremities and axial spine. It is surrounded by numerous muscles and ligamentous structures, which stabilize the joint and allow it to perform this critical function. The ventral 1/3 of the joint is hyaline cartilage and creates a true synovial joint, while the posterior 2/3 of the SIJ is bound by the interosseous sacroiliac ligament and posterior sacroiliac ligament, which are fibrocartilages [6, 7]. Histological studies performed on cadavers have confirmed the presence of nociceptive fibers within the intra-articular region [8], with posterior innervation of the joint originating mostly from the S1–S3 nerves [9]. The surface of the SIJ is covered with macroscopic irregularities at baseline, which interlock and provide stability. As patients age, arthritic pathological changes develop, worsening friction within the joint and creating instability [10]. These arthritic changes may likely complicate accessibility to the 1-2 mm joint space in patients [11].

### 1.2. Far-Contralateral Oblique (FCO) Approach

**Figure 1:**
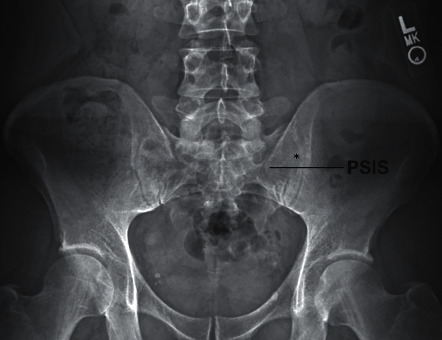
Radiographic AP view of the pelvis illustrating location of PSIS. Note the PSIS position in relation to the needle entrance point (^*∗*^) when utilizing FCO technique.

Accessing the middle third of the sacroiliac joint using the traditional 10 to 15° contralateral oblique angle can be challenging due to the presence of the posterior superior iliac spine (PSIS) (Figure 1).

**Figure 2:**
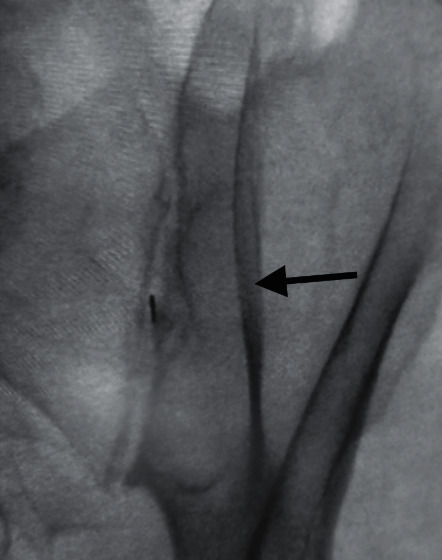
Contralateral oblique until PSIS is lateral to the SIJ line (black arrow).

**Figure 3:**
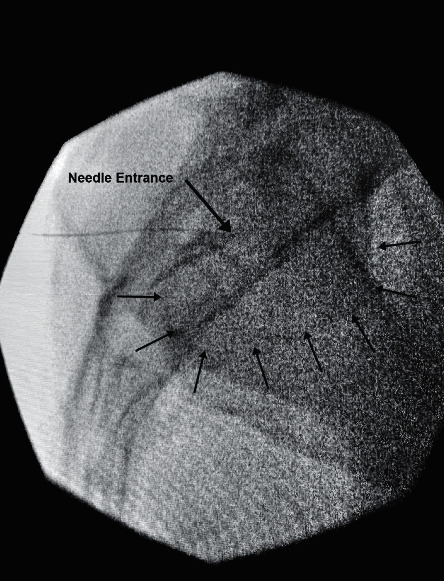
Lateral view of FCO needle placement (large arrow) with arthrogram enhancement (small arrows).

A far-contralateral oblique angle (typically between 20 and 40 degrees) can be advantageous for 2 reasons: to position the PSIS away (Figure 2) from the SIJ to gain access and to line up the anterior and posterior SIJ line. This can be accomplished in a few easy steps (Figure 3):

1. Take an AP fluoroscopic image over the side of the SIJ being injected to identify the PSIS.
2. Oblique the fluoroscopic image until the anterior and posterior aspects of the SIJ are in line. The degree of obliquity should place the cortical edge of the PSIS lateral to the SIJ.
3. Insert the spinal needle in the same trajectory as the fluoroscopy over the middle third of the SIJ between S1 and S2.
4. Use tactile input to feel needle entering the SIJ space.
5. Lateral fluoroscopic imaging should be used to confirm that the spinal needle is within the middle third of the SIJ and at least midway to the anterior border of the sacrum.
6. Ideally inject <1 mL of contrast to confirm intra-articular placement.

### 1.3. Conventional Sacroiliac Joint Injection Technique

**Figure 4:**
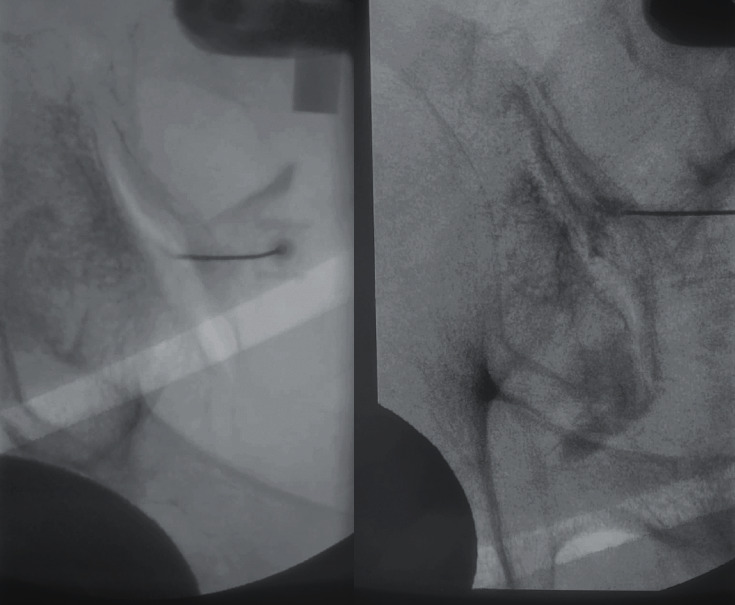
FCO view with needle entering from medial to lateral along mid-body of the left sacroiliac joint (left). AP view with sacroiliac arthrogram (right).

Injection techniques of the sacroiliac joint have been described since the 1990s. Historically, sacroiliac joint injections were performed without image guidance. The adoption of image guidance (CT, ultrasound, and fluoroscopy) has been accepted universally. The conventional technique of SI joint injections dictates aligning the anterior and posterior aspect of the joint under fluoroscopic guidance by giving 10 to 20 degrees of contralateral oblique angulation. This leads to visualization of the anterior and posterior joint as a single line at the inferior most portion of the joint [12]. The technique has the advantage of allowing access of the sacroiliac joint with short distance from the skin, eliminating the ileum from the trajectory of the needle, and allowing for a reproducible procedure (Figure 4).

Correct needle placement is confirmed by injecting a radiopaque contrast medium into the joint. At times it may be difficult to align the anterior and posterior joint fluoroscopically. Furthermore, there may be times where the inferior sacroiliac joint space is not accessible despite optimal fluoroscopic positioning. This can be due to several issues including anatomical variance, advanced spondylosis, and degenerative change.

There have been different techniques described with alterations in the angulation of imaging and various entry sites to the sacroiliac joint [13–16], although broad adoption of these techniques has been elusive.

In one technique described, the conventional method of entering the posterior joint was used. However, fluoroscopy was used in a true-anteroposterior (AP) view. In the case series, sixty (60) sacroiliac joints were injected, with only four (4) joints showing incorrect contrast spread [17]. The authors concluded that aligning the anterior and posterior aspects of SI joint for fluoroscopic-guided SI joint injection was not necessary. In a comparative study, thirty patients were randomized into 2 groups of 15 patients each. The endpoints measured included the total length of procedure time, fluoroscopic time, needling time (length of time the needle was maneuvered), and pre and post-procedure visual analogue scale pain scores [18]. This study of the posteroanterior approach for fluoroscopic-guided sacroiliac joint injection observed shorter times for fluoroscopy, needling, and the overall procedure than those recorded for the widely prevalent oblique approach.

In another study, the conventional approach was initially used, and once the needle had reached the target zone, the oblique views (ipsilateral and contralateral) were used to ensure that the needle is placed within the joint space. Then, the position of the needle tip was checked using lateral fluoroscopy. Out of thirty cases, 27 were reported to have satisfactory contrast spread [14].

Practice guidelines have previously referenced a superior approach, but only more recently described that in twenty-four patients [19]. Pain scores and disability were significantly reduced at 2 weeks and 4 weeks after treatment. Nineteen patients (79%) reported satisfaction with treatment. The wedge shape formed by the medial border of the ilium and the lateral border of the sacral ala was targeted under C-arm fluoroscopy guidance in a 40°–50° contralateral oblique view.

Kurosawa et al. described an intra-articular injection technique to the middle portion of the sacroiliac joint [20]. This procedure best coincides with the far-contralateral oblique (FCO) approach; however, there are some slight differences. The main difference is that the needle entry site is caudal to the PSIS. In order to accommodate this, a caudal tilt of 25–30° to the fluoroscope was utilized. In the 100 sacroiliac joint injections (69 consecutive patients), the authors reported a 80% success rate for entering the joint. The middle portion technique failed in cases of extremely narrow recess spaces and twisting at the recess line just in front of the posterior joint line.

### 1.4. Technique Pearls

1. The PSIS still may obstruct the SIJ even in far-contralateral oblique angles. One solution would be to use a cephalad tilt to remove the PSIS and guide the needle from superior to inferior into the SIJ space.
2. Guide the needle more medial to the SIJ than lateral. If the needle encounters the PSIS, it is far easier to correct medial than it is lateral.
3. A longer spinal needle may be necessary—with the increased degree of obliquity and cephalad tilt, there is a resultant increased distance within the soft tissue.

## 2. Conclusion

As interventions for sacroiliac joint dysfunction continue to grow, ensuring appropriate access into the joint itself is paramount. Placing a needle in sacroiliac joint via its most caudal access point has been widely adopted. Still the use of various techniques is widely practiced. The FCO approach is one of the latest techniques to be proposed in accessing the sacroiliac joint space and can be utilized to determine if the patient is a candidate for posterior percutaneous SIJ fusion. In conclusion, a novel FCO technique has been described in this article that can be used as an alternative to conventional SIJ technique. Further studies are necessary to determine the safety and effectiveness of the FCO technique.
